# The effect of titanium dioxide synthesis technique and its photocatalytic degradation of organic dye pollutants

**DOI:** 10.1016/j.heliyon.2018.e00681

**Published:** 2018-07-17

**Authors:** David Dodoo-Arhin, Frederick Paakwah Buabeng, Julius M. Mwabora, Prince Nana Amaniampong, Henry Agbe, Emmanuel Nyankson, David Olubiyi Obada, Nana Yaw Asiedu

**Affiliations:** a Department of Materials Science and Engineering, University of Ghana, P.O. Box Lg 77, Legon, Accra, Ghana; b African Materials Science and Engineering Network (A Carnegie-IAS RISE Network), Ghana; c Department of Physics, University of Nairobi, P.O. Box 30197-00100, Nairobi, Kenya; d INCREASE (FR CNRS 3707), ENSIP, 1 rue Marcel Doré, TSA41105, 86073 Poitiers Cedex 9, France; e Centre Universitaire de Recherche sur l'Aluminium, Université du Québec à Chicoutimi, Québec G7H 2B1, Canada; f Department of Mechanical Engineering, Ahmadu Bello University, Zaria, Nigeria; g Department of Chemical Engineering, Kwame Nkrumah University of Science and Technology, Kumasi, Ghana

**Keywords:** Materials science

## Abstract

Nanostructured mesoporous titanium dioxide (TiO_2_) particles with high specific surface area and average crystallite domain sizes within 2 nm and 30 nm have been prepared *via* the sol-gel and hydrothermal procedures. The characteristics of produced nanoparticles have been tested using X-Ray Diffraction (XRD), Brunauer–Emmett–Teller (*BET*) surface area analysis, Scanning Electron Microscopy (SEM), Fourier Transform Infra-Red (FTIR), and Raman Spectroscopy as a function of temperature for their microstructural, porosity, morphological, structural and absorption properties. The as-synthesized TiO_2_ nanostructures were attempted as catalysts in Rhodamine B and Sudan III dyes' photocatalytic decomposition in a batch reactor with the assistance of Ultra Violet (UV) light. The results show that for catalysts calcined at 300 °C, ∼100 % decomposition of Sudan III dye was observed when Hydrothermal based catalyst was used whiles ∼94 % decomposition of Rhodamine B dye was observed using the sol-gel based catalysts. These synthesized TiO_2_ nanoparticles have promising potential applications in the light aided decomposition of a wide range of dye pollutants.

## 1 Introduction

With the rapid growth in industrialization, a considerable amount of waste keeps being discharged into the environment. These on-going waste disposal processes tend to compromise the quality of water for daily use despite the great importance of clean water to society [1, 2, 3]. Dye pollutants from the textile, dye, food, leather and paper industries, among others, contaminate the environment when they are improperly disposed-of [4, 5, 6, 7, 8]. Yet these synthetic polymeric dyes have a wide spectrum of applications, which makes their use inevitable. The only way to deal with these dye pollutants is to develop safe disposal mechanisms and methodologies so that they can co-exist with the ecosystem to provide a green environment. To this end, a plethora of waste water treatment strategies such as neutralization by acid and basic liquors, chemical oxidation and flocculation, activated and effluent with loaded organic carbon, biological degradation means and photocatalysis have all been investigated [9]. Among the aforementioned strategies, photocatalysis appears most appropriate, as it is energetically sustainable, eco-compatible and gives a total or partial decomposition of dye pollutants. In particular, different classes of dyes have been utilized in modelling organic contaminants in effluents of wastewater. Recently conducted surveys of literature have shown that out of 12,400 papers recorded in the Web of Science that are devoted to photocatalysis, just a small percentage of dyes have been investigated; with Methyl Orange (36 %) and Methylene Blue (47 %) receiving the most attention [10]. Though the remaining 17 % may arguably be Azo dyes, it appears Sudan III (a neutral class of azo dye) is rarely reported. Azo dyes are characterised by their N=N chromophoric unit in their molecular structures [11]. Since they constitute approximately 60–70 % of all available commercial dyes products used in the textile industry [12], they are worthy of investigation. Aarthi, T. et al [13], have reported the effect of functional groups in Sudan (III and IV) and Azure (A and B) Azo dyes on the structure and degradation rate using TiO_2_ nano-photocatalysts. They demonstrated that the degradation rate of these dyes was affected by their molecular structures. The same grouped also provided an excellent work on molecular structure-degradation rate relations for Xanthene class of dye such as Rhodamine [14].

TiO_2_ nanostructures represent a promising semiconductor for several applications such as Solar cells [15], batteries [16], sensors [17] and catalytic degradation of dye pollutants owing to their chemical stability, strong oxidizing ability, non-toxicity, mechanical robustness, low cost and efficient photocatalytic activity [18]. It is well documented in literature that, the photocatalytic effectiveness of TiO_2_ is intensely related to its structural, morphological and textural properties. This has led to the development and synthesis of a myriad of TiO_2_ possessing different structures and morphologies. The degradation of dye pollutants using TiO_2_ as catalyst involves the exposure of the catalytic material to ultra violet light, which is often linked with the creation of holes in the valence and electrons in the conduction bands responsible for oxidizing and reduce the dye pollutants. Nonetheless, critical issues that often limit the photocatalytic activity of TiO_2_ materials are the wide band gap energy (3–3.2 eV), low mass transport rates and low quantum yield. To succumb these critical challenges, a plethora of scientific strategies have been adopted by researchers, such as reducing grain size of TiO_2_ and increasing its specific surface area and also by employing different synthesis techniques such as solvothermal method [19], precipitation method [20], sol-gel method [21], thermal decomposition of alkoxide [22], chemical vapor deposition [23]. Among these techniques, sol–gel technique, is a simple, economical, convenient and accomplished technique for producing nanoparticles which have different morphologies such as sheets, tubes, quantum dots, wires, rods and aerogels. The hydrothermal technique also produces mono-dispersed and highly homogeneous nanoparticles due to the highly controlled diffusion in the crystallization medium and phase transformations at relatively low temperature [24]. To the best of our knowledge, a comparative investigation on sol-gel synthesis and hydrothermal synthesis methods on degradation of Azo dyes (Sudan III) and Xanthene dye (Rhodamine B) is yet to be reported in the literature.

In this research work, we investigate the influence of synthesis method (sol-gel and hydrothermally synthesis) of TiO_2_ nanoparticles on the light-aided catalytic decomposition of Rhodamine B and Sudan III dyes under varied durations of UV illumination. The influence of annealing temperatures on the surface area of the synthesized TiO_2_ samples was also investigated. Several characterization techniques were further used to investigate the structural, thermal, optical and crystallinity of the synthesized TiO_2_ samples.

## 2 Experimental

In this research, we used analytically rated chemicals reagents which were not further purified. These chemical reagents used were Rhodamine B dye (C_28_H_31_ClN_2_O_3_), Sudan III dye (C_22_H_16_N_4_O), distilled water, Titanium tetrachloride (TiCl_4_), Titanium isopropoxide (TTIP), isopropyl alcohol (IPA), ethanol.

Sol gel and hydrothermal methods were adopted to synthesize TiO_2_ nanoparticles by using different metal alkoxide precursors.

In the typical sol-gel synthesis process, the precursor solution consisting of (TiCl_4_) and absolute ethanol in the ratio of 1: 10 were vigorously stirring at 1200 rpm for 4 hrs at room temperature to enhance the homogeneity and stability of the slurry. The sol formed was allowed to age under room temperature conditions for 18 hrs, sonicated for 30 min and dried in an oven at 120 °C for 7 hrs. The prepared powder was then subjected to elevated temperatures of calcination at 200 °C, 300 °C, 400 °C, 500 °C, and 600 °C for 2 hrs to obtain the TiO_2_ nanoparticles for analysis.

In the hydrothermal technique, 10 mL of TTIP was added to 100 mL of isopropyl alcohol under constant magnetic stirring at 1200 rpm for about 10 min for homogeneity. 10 mL of distilled water was added slowly at a rate of 2 mL/min and the solution was subsequently stirred at 1200 rpm for 10 min. The resulting solution was then aged at room temperature conditions for 24 hrs. After sonicating the sol for 10 min it was moved to a teflon-lined steel autoclave under 15psi, 120 °C for 8 hrs. After cooling down to room temperature, the as-prepared TiO_2_ nanoparticles were calcined at 200 °C, 300 °C, 400 °C, 500 °C and 600 °C for 2 hrs to obtain the TiO_2_ nanoparticles for further characterization and application.

The obtained TiO_2_ nanoparticles are then tested using the Fourier Transform Infra-Red (FT-IR) spectroscopy, X-Ray Diffraction (XRD), Brunauer–Emmett–Teller (BET) surface area analysis and Scanning Electron Microscopy (SEM) to determine the crystal structural, microstructural, surface and porosity properties. For the qualitative phases determination as well as the microstructure of the produced TiO_2_ powders, powder x-ray diffraction (PXRD) profiles were collected using a Panalytical Empyrean diffractometer having a theta-theta geometry and equipped with a copper (wavelength = 1.5418 Å) radiation tube operated at a voltage of 40 kV and current of 45 mA. The collection of the diffraction profiles of all the powder samples were done within 20°–70° at a step size of 0.017° and 14 seconds per step. The qualitative phase evaluation of diffraction profiles conducted using the X'Pert Highscore plus search match software (Panalytical, Netherlands) and matched with the ICSD's powder diffraction files database. To aid the microstructure profile fitting analysis, the resolution contribution function for the diffractometer was determined using the NIST SRM 640d (Si) standard [25] after which and the resultant peak profiles were concurrently fitted by applying shape and width restricted symmetrical pseudo-Voigt functions as prescribed by the Caglioti formulae [26]. Subsequently, the microstructural determination was accomplished via the Whole Powder Pattern Modelling (WPPM) technique [27], using the software called PM2K [28].

For the morphological investigations, the nanoparticles were sputtered coated with carbon to make the particles conductive and allow for higher magnifications in a high resolution Zeiss Ultra plus 55 field emission scanning electron microscope (FESEM) which was operated at 2.0 kV. The structural properties where determined on a Jobin Yvon Horiba TX 6400 micro-Raman spectrometer possessing a triple monochromator system and a LabSpec (Ver. 5.78.24) analytical software. All the powder samples were analysed using an Argon excitation laser (514 nm) through an X 50 objective piece with a resolution of 2 cm^−1^ and an acquisition time of 120 seconds. Prior to the BET surface area and porosity evaluations, about 0.2 g the powder samples were degassed around a temperature of 300 °C under vacuum conditions for 24 hrs. The information from the adsorption-desorption isotherms were used for the Brunauer-Emmett-Teller (BET) Surface Area evaluation and Barrett-Joyner-Halenda (BJH) Pore Size-Volume Investigation respectively [29]. Transmission FT-IR spectroscopic data were collected within a range of 4000–400 cm^−1^ and a resolution of 4 cm^−1^ using a Bruker Vertex 70v FTIR spectrometer equipped with an evacuatable sample compartment, a diamond ATR crystal (*2 mm sample-crystal contact diameter*) and an Opus acquisition software for analysis.

The photocatalytic activity of the titanium dioxide photocatalysts were evaluated by determining the decomposition of Rhodamine B and Sudan III dyes under the irradiation of Ultra Violet light (UV). The experiments were carried out in a 100 mL capacity double-walled quartz glass immersion wells photochemical reactor (technistro) equipped with a chiller, reaction flask to lamp distance of 20 mm, and a 330 Watt medium pressure mercury lamp (UV lamp, 365 nm). In a typical experimental procedure, Rhodamine B dye (0.01 g) was mixed with 200 mL of distilled water to obtain the dye solution. 0.1 g of the as-prepared TiO_2_ nanoparticles was mixed with 50 mL of distilled water in a different beaker to obtain the TiO_2_ suspension. 30 ml of the dye solution was then added to the prepared TiO_2_ suspension. Prior to UV irradiation, the suspension was stirred in the dark room at 1000 rpm for 30 minutes for optimum adsorption. After the 30 min stirring in the dark room, it was then exposed to a 330 W UV light source. While this suspension was exposed to the UV light, 5 mL was withdrawn with syringe every 30 min. The aliquot was centrifuged at 5000 rpm for 10 min. The supernatant was transferred into a cuvette and analyzed using a Pelkin-Elmer Lambda 850 UV-vis spectrophotometer to determine the absorption over a wavelength range of 200 nm–800 nm. The various exposure times observed in this work were 0, 30, 60, 90, 120 and 150 min. The concentration of the Rhodamine B dye in solution was decreased and the photocatalytic procedure repeated as described above. Here, a mixture was obtained by mixing 0.01 g of the Rhodamine B dye with distilled water (400 mL) to obtain the dye solution. 0.1 g of the powder was also mixed with about 50 mL of distilled water in a different beaker to obtain the TiO_2_ suspension. This was followed by the addition of 10 mL of the Rhodamine B dye solution to the 50 mL TiO_2_ suspension. Prior to UV irradiation, the suspension was stirred in the dark room at 1000 rpm for 30 min. After the 30 min of stirring in the dark room, it was then exposed to a UV light operating at 330 W power. Optical UV-Vis spectra were taken at different exposure times. The parameters involved in the photocatalytic degradation of Rhodamine B dye solution are summarized in Table 1.

**Table 1:** Rhodamine B dye degradation parameters.

Concentration of Rhodamine B dye solution		pH	Volume of Rhodamine B dye solution	Mass of TiO_2_ catalyst	Method of catalyst preparation
(mol/dm^3^)	(ppm)		(ml)	(g)	
3.8 × 10^−5^	18.2	3.5	80	0.1	Sol gel
8.5 × 10^−6^	4.1	4.0	60	0.1	Sol gel
3.8 × 10^−5^	18.2	3.5	80	0.1	Hydrothermal
8.5 × 10^−6^	4.1	4.0	60	0.1	Hydrothermal

In the Sudan III dye degradation experiment, a stock solution was prepared by dissolving 0.8 g Sudan III dye in 400 mL Isopropyl alcohol under sonication for about 30 min. The solution was filtered and the filtrate used as the dye solution for the photodegradation experiment. 50 mL of the as-prepared Sudan III dye solution was mixed with 0.1 g of TiO_2_ catalyst to obtain Sudan III – TiO_2_ suspension. Prior to UV irradiation, the mixture was then stirred in the dark at 1000 rpm for about 30 min to ensure uniform mixture. It was then exposed to a 330 W UV light irradiation. While this suspension was exposed to the UV light, 5 mL aliquot was withdrawn with a syringe every 30 min over a 150 min exposure period. Each aliquot was centrifuged at 5000 rpm for a period of 10 min and the supernatant transferred into a cuvette for absorption analysis on a Pelkin-Elmer Lambda 850 UV-Vis spectrophotometer over a wavelength range of 200 nm–800 nm. Batch formulations with reduced dye concentrations were analyzed under varying exposure times following the above discussed procedure. The parameters involved in the photocatalytic degradation of Sudan III dye solution are summarized in Table 2.

**Table 2:** Sudan III dye degradation parameters.

Concentration of Sudan III dye solution		pH	Volume of Sudan III dye solution	Volume of Isopropanol	Mass of TiO_2_ catalyst	Method of catalyst preparation
**(mol/dm**^3^**)**	(ppm)		(ml)	(ml)	(g)	
5.7 × 10^−3^	2008.7	8.3	50	0	0.1	Sol gel
2.3 × 10^−3^	810.5	4.5	20	30	0.1	Sol gel
5.2 × 10^−4^	183.3	4.0	5	50	0.1	Sol gel
5.7 × 10^−3^	2008.7	8.3	50	0	0.1	Hydrothermal
2.3 × 10^−3^	810.5	4.5	20	30	0.1	Hydrothermal
5.2 × 10^−4^	183.3	4.0	5	50	0.1	Hydrothermal

## 3 Results and discussion

In the synthesis of TiO_2_ nanoparticles by hydrothermal techniques, alkoxides were hydrolysed in the presence of water and subsequently polymerized to obtain a three-dimensional oxide network. A colourless solution was obtained when the Titanium tetraisopropoxide (TTIP, Ti(OCH(CH_3_)_2_)_4_) solution was reacted with isopropanol (IPA), but a white precipitate was formed when water was added dropwise. The reactions are presented as follows in Eqs. (1), (2), and (3), starting from the reactions between the Titanium alkoxide (TTIP and IPA) and water. Eqs. (1) and (2) show the hydrolysis and condensation reactions respectively. Calcining the samples at various temperatures gave the resultant TiO_2_ nanoparticles as illustrated by Eq. (3).

In the Sol gel processing of TiO_2_ nanoparticles, the reaction kinetics are described in Eqs. (4) and (5). The hydrolysis reaction was initiated when the Titanium tetrachloride precursor was reacted with absolute ethanol to form the sol. In this process hydrochloric acid gas was seen expelled from the reaction mixture and a yellow suspension was seen after 2 hours. After the Titanium organic compound obtained was heat treated, white powdered TiO_2_ nanoparticles were obtained as the hydrocarbon components were expelled through the heat treatment process.

(1) $$Ti{\left(OCH{\left(CH_{3}\right)}_{2}\right)}_{4}+4H_{2}O\;\to Ti{\left(OH\right)}_{4}+4{\left(CH_{3}\right)}_{2}CHOH$$

(2) $$\mathrm{Ti}{\left(\mathrm{OH}\right)}_{4}\to \mathrm{Ti}O_{2}\cdot xH_{2}O+\;\left(2-x\right)H_{2}O$$

(3) $$\mathrm{Ti}O_{2}\cdot xH_{2}O\;\overset{\mathrm{heat}}{\to }\;TiO_{2}$$

(4) $$\mathrm{TiC}l_{4\;}+4C_{2}H_{5}OH\;\to \mathrm{Ti}{\left(C_{2}H_{5}O\right)}_{4}+4\mathrm{HCl}$$

(5) $$\mathrm{Ti}{\left(C_{2}H_{5}O\right)}_{4}\overset{\mathrm{heat}}{\to }\;TiO_{2}+\mathrm{hydrocarbons}$$

The titania nanoparticles were heated at different temperatures and analyzed by X-ray diffraction (XRD). Whole Powder Profile Modelling using PM2K software was used to analyze the TiO_2_ nanoparticles prepared. Grain sizes and crystalline phases of the catalyst samples were derived from analysis of the XRD patterns [30].

**Fig. 1:**
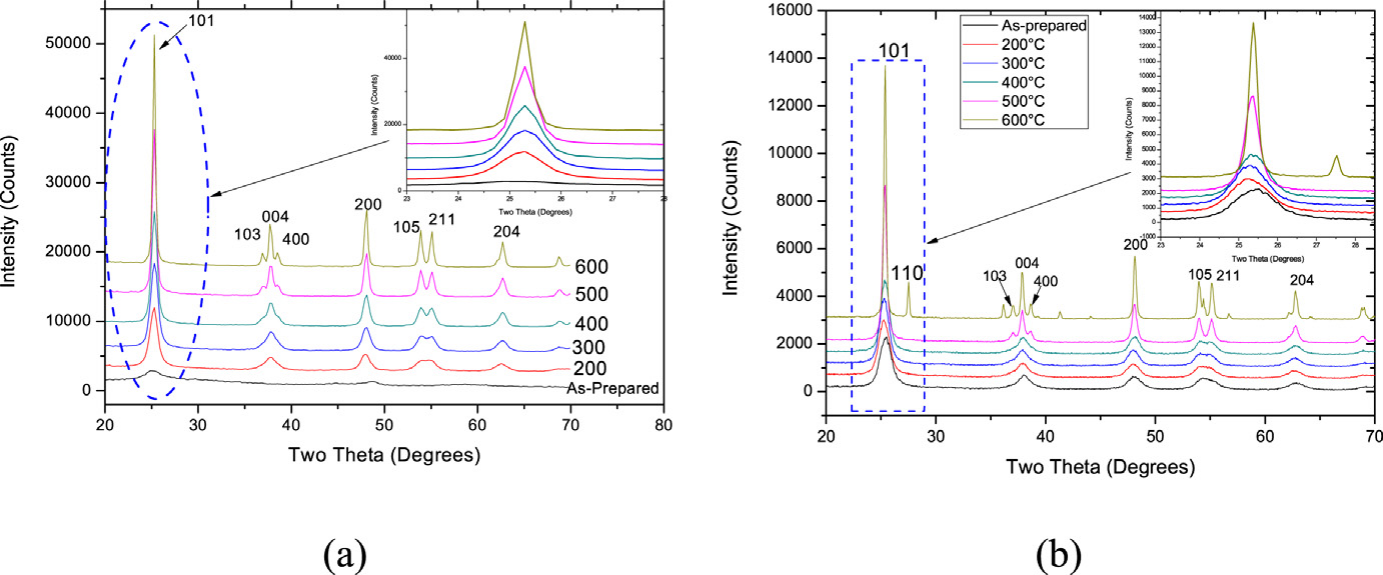
XRD Pattern (a) TiO_2_ nanoparticles prepared by sol gel method (b) TiO_2_ nanoparticles prepared by hydrothermal method.

The XRD patterns (Fig. 1a) of the TiO_2_ powders synthesized using the sol-gel technique, showed that the anatase phase of TiO_2_ nanoparticles was produced. Hence, calcination tends to improve the crystallinity of the nanoparticles which consequently transforms amorphous titanium dioxide (TiO_2_) into its anatase phase, which upon further increase in the calcination temperature, transforms into the rutile phase. This phenomenon is consistent with available literature which tend to indicate that, anatase-rutile phase transitions typically occurs between 450 °C and 600 °C: the variation in the transition temperatures is influenced by the type of precursors used, the synthesis conditions and the characteristics of the produced particles [31]. Fig. 1a shows that when the heating temperature increases from 200 °C to 600 °C, there is an increase in the peak intensities as well as narrowing of the peak width, suggesting the transformation of the amorphous as-prepared TiO_2_ catalyst to a highly crystalline TiO_2_ samples. It's noteworthy that, for the sol-gel produced particles, no anatase-rutile phase transition was observed, no matter the calcination temperature used within our investigated temperature range. This demonstrated that, the titanium dioxide powders prepared using the sol-gel technique had relatively high thermal phase stability, which tends to subdue the phase transition from anatase to rutile. Contrary to the XRD results of the titania samples prepared using the sol-gel method, the XRD analysis of the titania samples prepared by hydrothermal method and calcined at 600 °C exhibited a mixture of rutile and anatase phase.

**Fig. 2:**
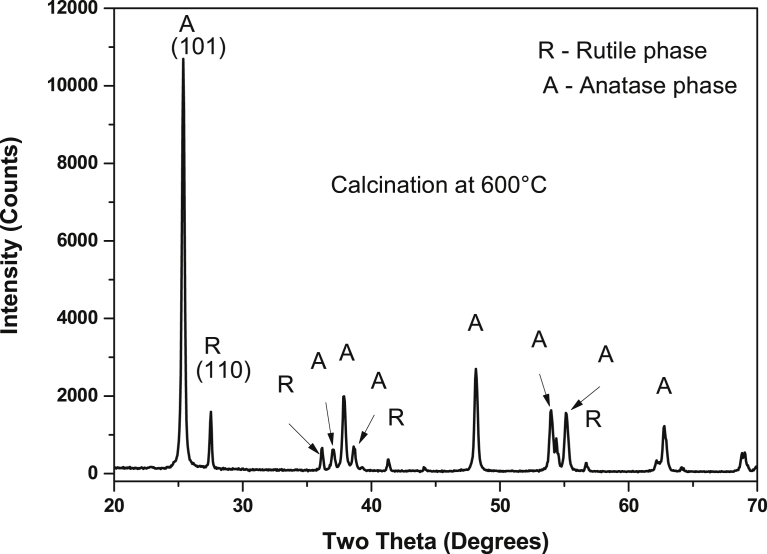
XRD TiO_2_ catalyst (synthesized by hydrothermal method) calcined at 600 °C.

When the calcination temperature was increased to 600 °C, a rutile phase, confirmed by the diffraction peak in [110] direction, which is a more stable phase at high temperatures, was seen (Fig. 2). This was observed as the peaks labelled **R**, whereas the Anatase phase direction is labelled **A**. It was expected that the rutile phase peaks would increase in intensity with increasing annealing temperature since temperature increases the stability of the rutile phases of TiO_2_.

The amount of anatase and rutile phases of TiO_2_ nanoparticles prepared by hydrothermal route and calcined at 600 °C were calculated using Eq. (1):

(6) $$X_{A}=\frac{100}{1+\left(1.265I_{R}/I_{A}\right)}$$

Where X_A_ is the weight fraction of the anatase phase, and I_A_ and I_R_ are intensities of the anatase (101) and rutile (110) diffraction respectively [32]. The TiO_2_ samples prepared via hydrothermal route and calcined at 600 °C resulted in 85 % and 15 % of anatase and rutile phase, respectively, according to Eq. (6). Nonetheless, at 200 °C–500 °C annealing temperatures, pure anatase phase of TiO_2_ was formed (Fig. 1).

**Fig. 3:**
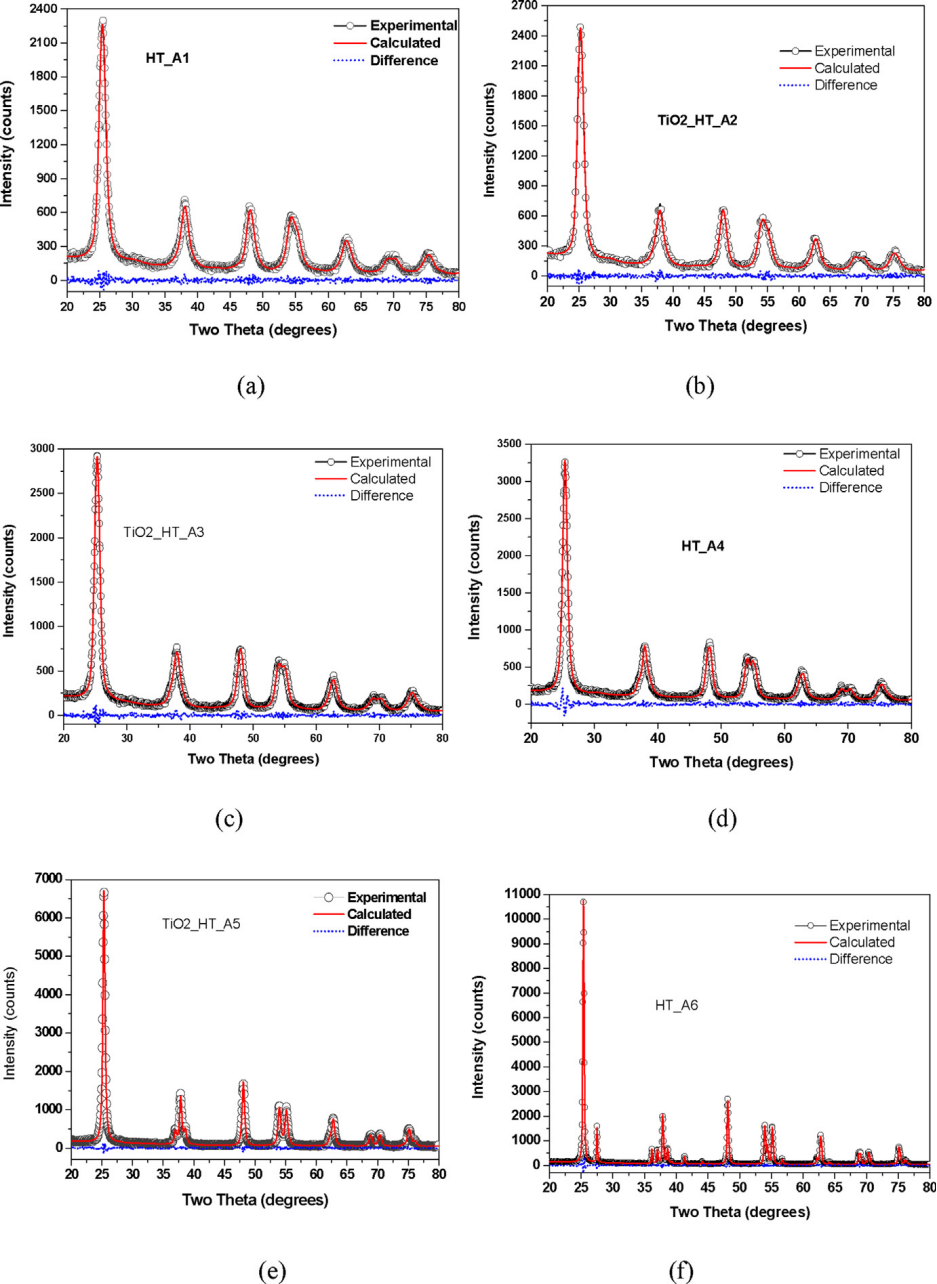
Modelled XRD of TiO_2_ nanoparticles synthesized by hydrothermal method (a) as-prepared (b) calcined at 200 °C (c) calcined at 300 °C (d) calcined at 400 °C (e) calcined at 500 °C (f) calcined at 600 °C.

**Fig. 4:**
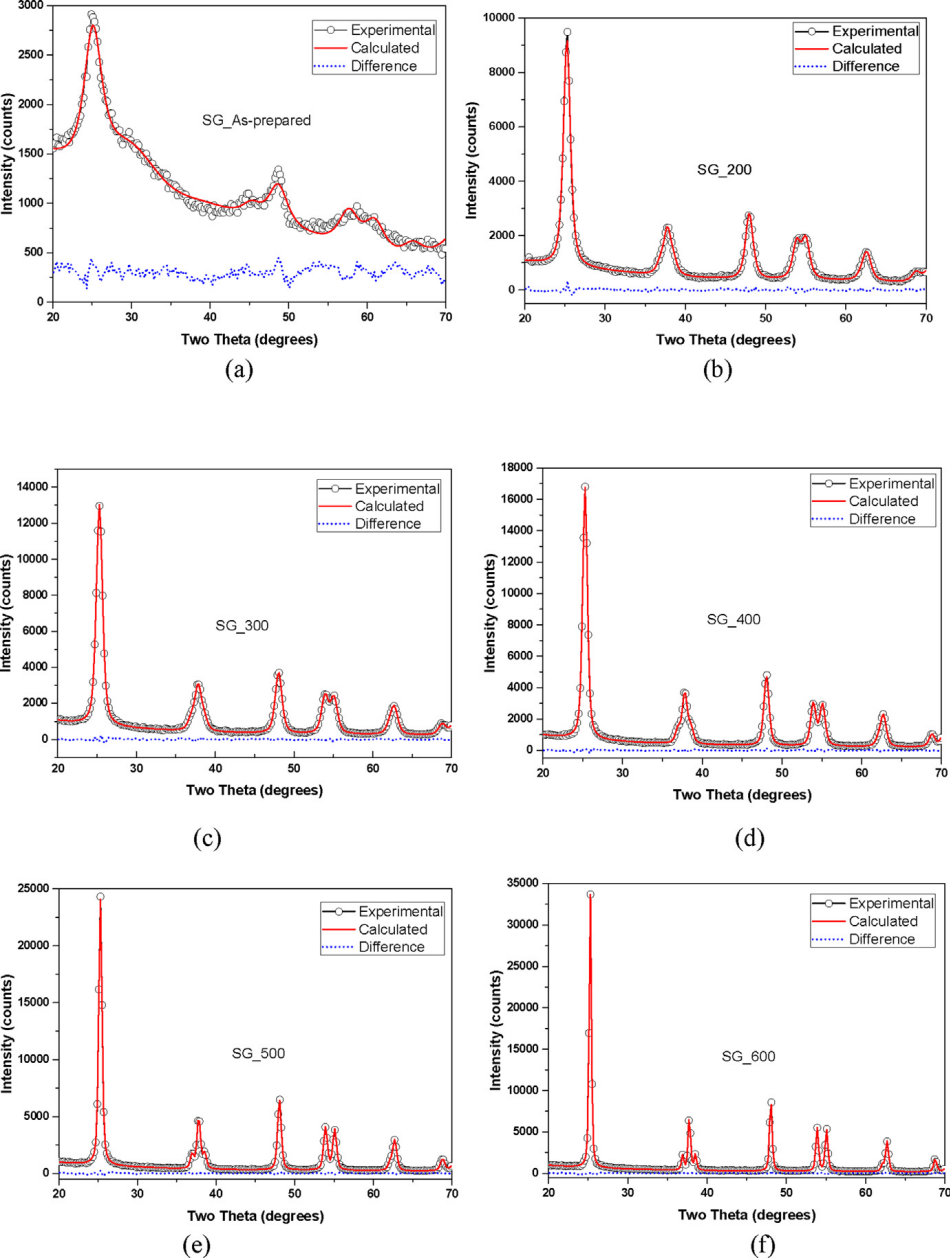
Modelled XRD of TiO_2_ nanoparticles synthesized by Sol gel method (a) as-prepared (b) calcined at 200 °C (c) calcined at 300 °C (d) calcined at 400 °C (e) calcined at 500 °C (f) calcined at 600 °C.

When the experimental XRD profiles were modelled using the Whole Powder Pattern Modelling (WPPM) technique incorporated in the PM2K software, a very low goodness of fit was obtained: an indication of a better fit between experimental and calculated data. The results of modelled XRD profiles are shown in Figs. 3 and 4, respectively.

**Fig. 5:**
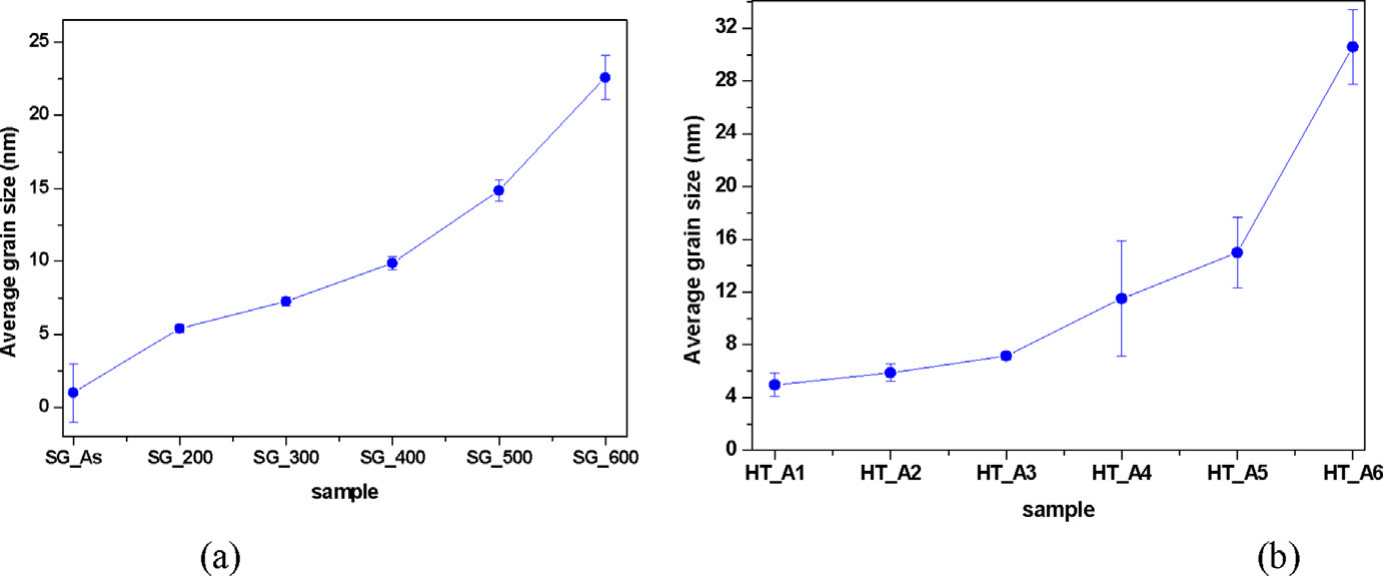
Average grain size as a function of temperature of TiO_2_ catalyst prepared by (a) Sol gel route and (b) Hydrothermal routes.

The grain sizes of the TiO_2_ nanoparticles synthesized by both hydrothermal and sol-gel techniques were investigated as a function of annealing temperature (Fig. 5a and b) respectively.

An average grain size of 2 nm was observed for the as-synthesized TiO_2_ nanoparticles synthesized using the sol-gel technique. Annealing the as-prepared TiO_2_ resulted in an increased grain size. More specifically, at 200 °C an approximate grain size of 5 nm was obtained, which further steadily increased upon elevation in calcination temperature. At 600 °C, a dramatic improvement in grain size (∼23 nm) was obtained possible as a result of the enhanced grain growth due to the high temperature treatment.

In the case of the TiO_2_ nanoparticles synthesized using the hydrothermal method, the as-prepared nanoparticles domain sizes were approximately 5 nm. The grain size increased gradually, from ∼6 nm at 200 °C to ∼15 nm at 500 °C. However, a two-fold increase in grain size (31 nm) was seen when the calcination temperature was raised to 600 °C. This observation could be attributed to grain growth as a result of increase in annealing temperature as well as the phase transition from the anatase phase to the rutile phase at 600 °C.

**Fig. 6:**
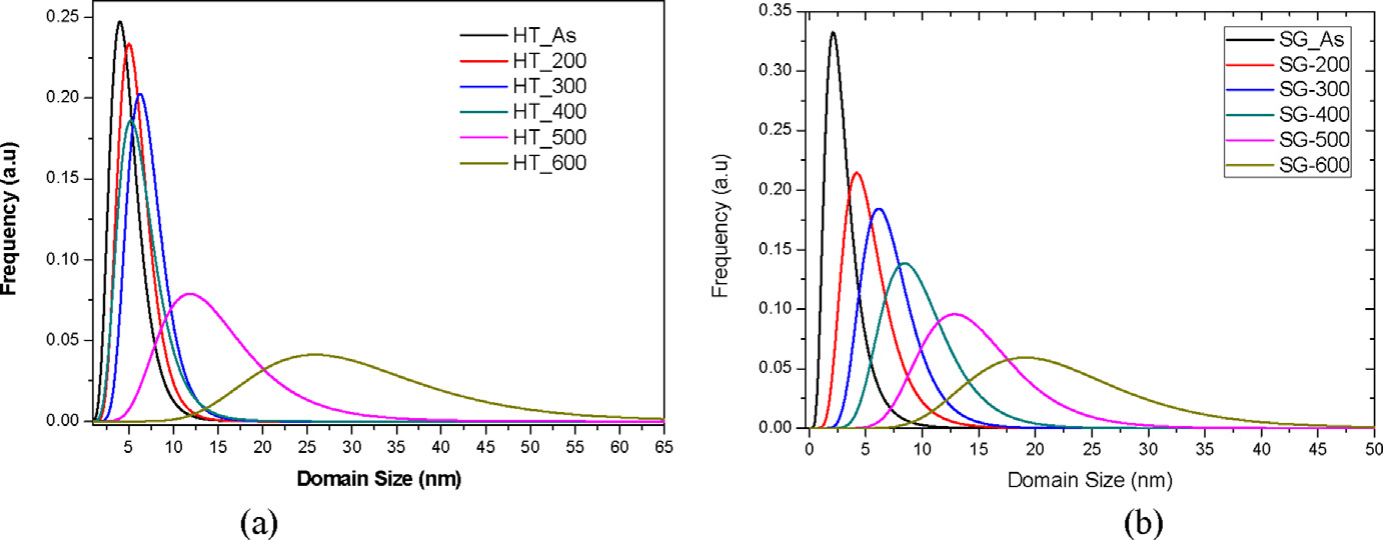
Lognormal size distribution of (a) Hydrothermal and (b) Sol-gel synthesized TiO_2_ nanoparticles.

Fig. 6 depicts the lognormal size distribution of TiO_2_ nanoparticles prepared *via* the hydrothermal technique. It shows clearly a narrow frequency peak around a 5 nm domain size for the as-prepared TiO_2_ nanoparticles**.**

The domain size increased and broadened slightly as the calcination temperature was raised up to 400 °C, with the domain size distribution revealed to be between 1 nm and 15 nm. However, the domain size increased and broadened drastically over a wide size distribution when the calcination temperatures were increased to 500 °C and 600 °C, respectively. It was therefore proven that there was a wide crystallite size distribution as well as a general increase in crystallite domain size as the temperature was increased.

The Lognormal size distribution of the nanoparticles synthesized using sol gel as shown in Fig. 6b also demonstrates a general widening of grain size distribution with increasing temperature. The peak of each distribution normally gives the average grain size of the synthesized TiO_2_ nanoparticles. It was also clearly demonstrated that the average grain size was increased as the heating temperature was increased. The as-prepared TiO_2_ nanoparticles which exhibited a highly amorphous nature showed an average grain size of 2 nm. The grain size increased gradually, and the size distribution broadened with increasing calcination temperature.

**Fig. 7:**
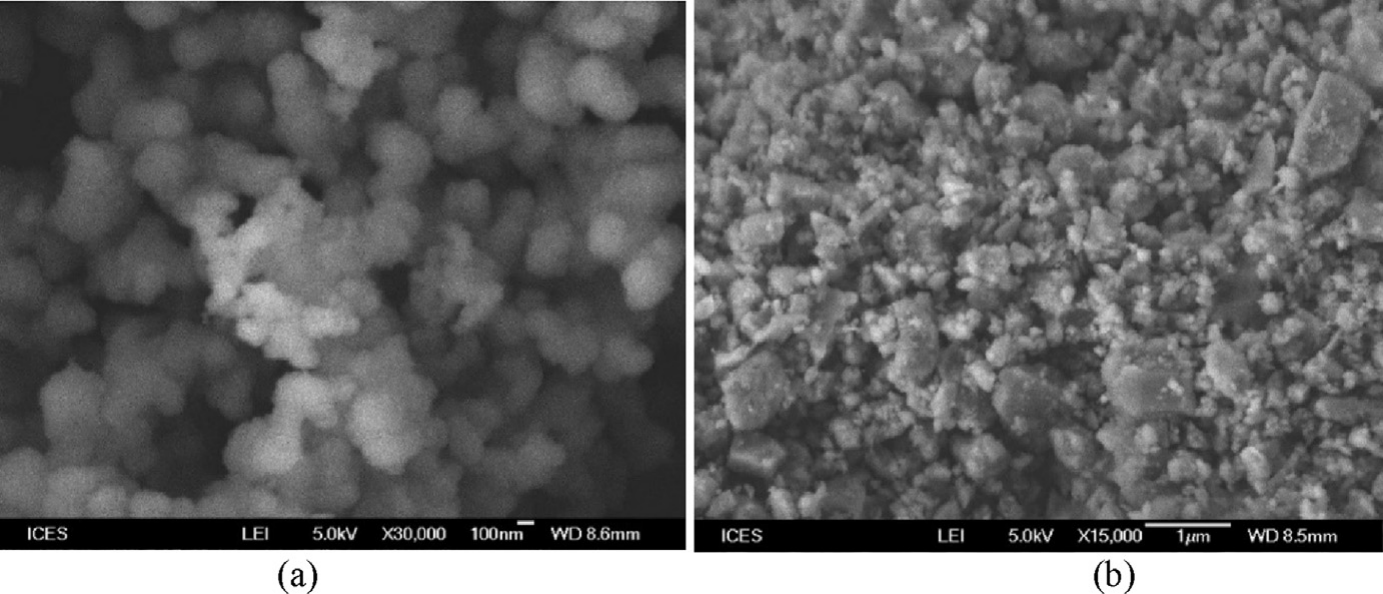
SEM images of calcined (300 °C) TiO_2_ nanoparticles prepared by (a) hydrothermal and (b) Sol-gel methods.

SEM analysis of the as-synthesized TiO_2_ nanoparticles demonstrated that nearly spherical nanoparticles were produced (Fig. 7).

**Fig. 8:**
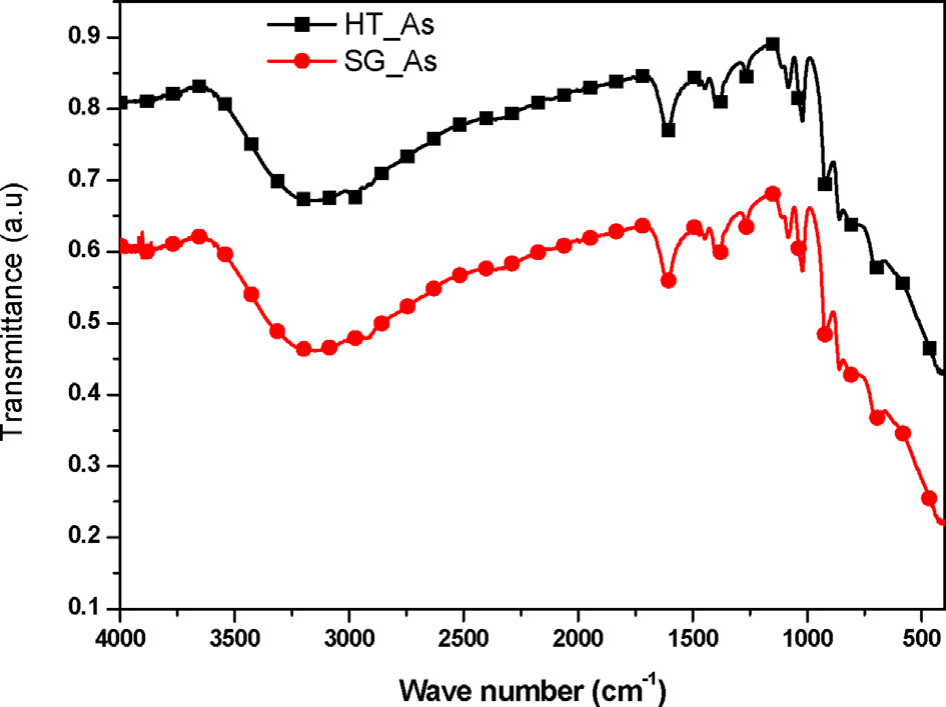
FTIR spectra of the prepared TiO_2_ nanoparticles.

FT-IR spectroscopy was also conducted on the synthesized nanoparticles to estimate the absorption of the incident light rays with respect to the wavelength of the incident beam. A representative FT-IR spectrum on the prepared TiO_2_ nanoparticles (Fig. 8) show a broad band around 3228 cm^−1^ which could be attributed to the O-H stretching mode of the surface and adsorbed water molecules.

The peaks centered at 1635 cm^−1^ were also attributed to the stretching of titanium carboxylate, which was formed from TiCl_4_ and ethanol precursors. The band between 800 cm^−1^ and 414 cm^−1^ was a result of the Ti-O bond stretching mode of the anatase polymorph of the TiO_2_ [32, 33].

**Fig. 9:**
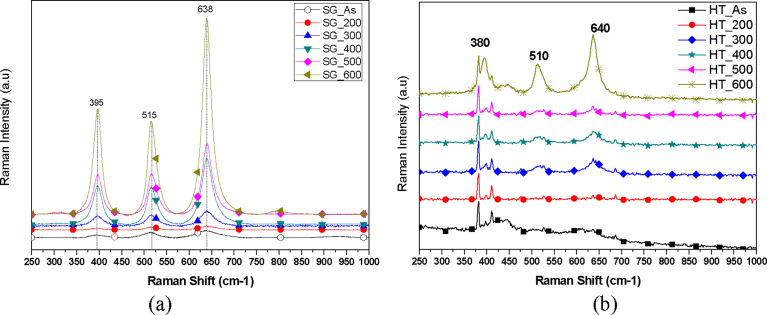
Raman Spectra of TiO_2_ nanoparticles synthesized by (a) sol gel method and (b) Hydrothermal method.

Further insight into the synthesized nanostructured TiO_2_ was gained by Raman spectroscopy. The TiO_2_ nanoparticles synthesized using the sol gel method showed intense Raman peaks at 395 cm^−1^, 515 cm^−1^ and 638 cm^−1^ (Fig. 9a).

The observed Raman spectra indicates that the TiO_2_ nanoparticles prepared and calcined at 200 °C, 300 °C, 400 °C, 500 °C and 600 °C were of anatase phase and in perfect agreement with the XRD results [34]. The Raman spectra shown in Fig. 9b revealed Raman peaks around 380 cm^−1^, 510 cm^−1^, and 640 cm^−1^ when the samples were prepared by the hydrothermal technique. Temperature treatment played an important role as it can be seen that the Raman peaks generally became more distinct and intense with increasing temperature. However, there was not a very significant difference in the Raman peaks as they increased gradually when the samples were calcined up to 500 °C.

The adsorption isotherms of nitrogen on TiO_2_ nanoparticles at an increasing relative pressure were analyzed to determine the BET surface area, pore size and pore volume. The adsorption-desorption isotherms did not seem to follow the same path for a definite range of relative pressures. This type of hysteresis loop is commonly exhibited in adsorbent materials which are mesoporous.

**Fig. 10:**
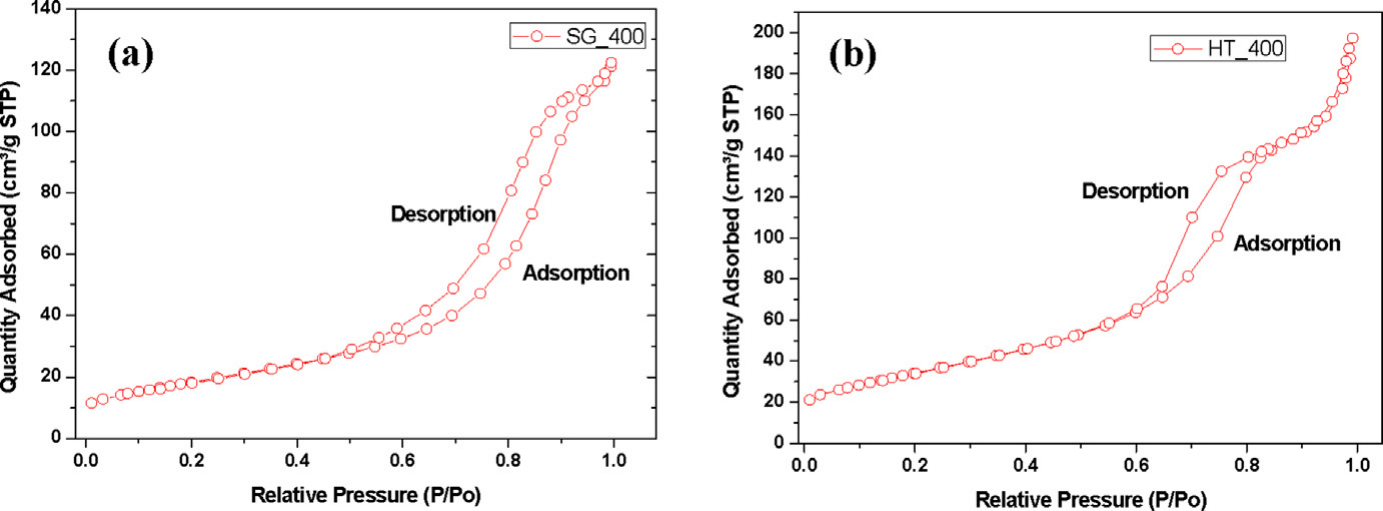
Isotherm linear plot of TiO_2_ nanoparticles synthesized by (a) Sol gel method and (b) Hydrothermal method.

From Fig. 10, the Linear Isotherm plot of the as-prepared TiO_2_ nanoparticles synthesized by sol gel method as well as samples calcined at 200 °C, 300 °C and 400 °C showed a stepwise adsorption-desorption branch at a wide range of pressure (P/Po): Type IV isotherm classification as prescribed by the IUPAC classification system [35]. This showed that mesoporous TiO_2_ nanoparticles were produced at these temperature treatments. At 500 °C and 600 °C calcination temperatures, a stepwise adsorption-desorption branch was not observed and the samples were classified as Type III isotherms according to IUPAC isotherm classifications. The linear isotherm plots of TiO_2_ nanoparticles prepared by hydrothermal method are presented in Fig. 10b. Isotherms exhibiting a characteristic type IV form and hysteresis loop, was observed for our as-prepared TiO_2_ nanoparticles as well as the samples heated at temperatures of 200 °C, 300 °C, 400 °C, and 500 °C: characteristic feature for materials with mesoporosity in accordance with the isotherm taxonomy of IUPAC. There was also an abrupt rise in volume of N_2_ adsorption that was observed and located in the P/P_o_ range of 0.6–0.8. This steady increase can be imputed to the capillary condensation, which indicates a good homogeneity of the sample and fairly small pore size since the P/P_o_ position of the inflection point is related to the pore dimension [36]. Sample calcined at 600 °C exhibited a Type III isotherm and in this case hysteresis loop with a stepwise adsorption-desorption branch was not observed [35].

**Fig. 11:**
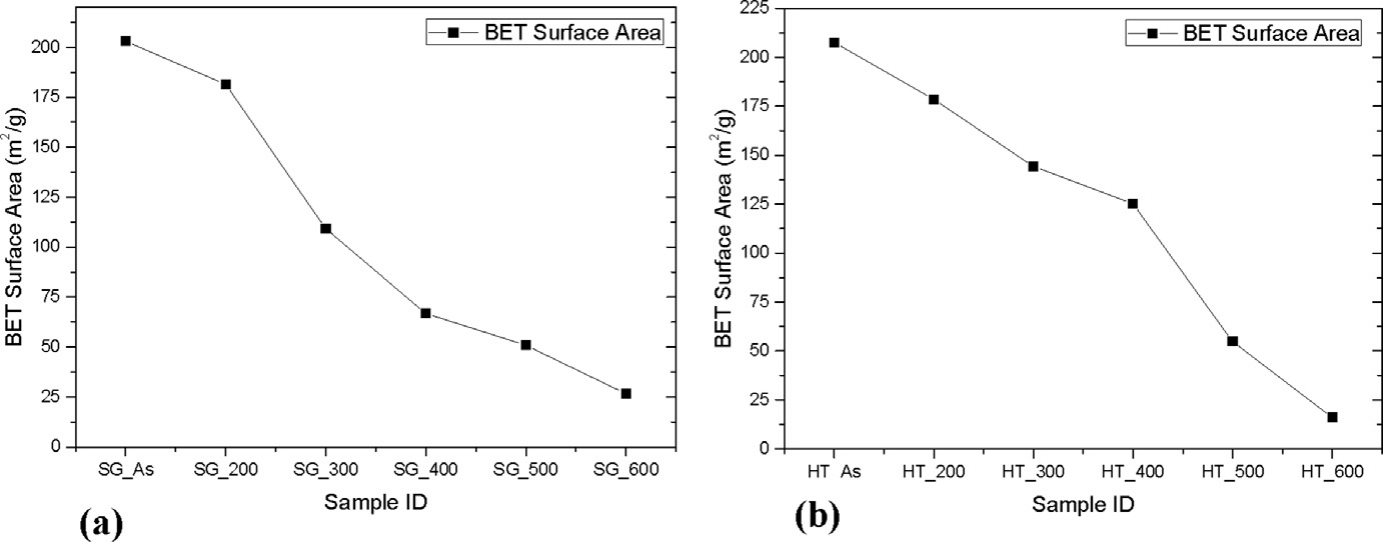
BET Surface area of TiO_2_ nanoparticles synthesized by (b) Sol gel method and (b) Hydrothermal method.

A plot of BET surface area against the different heating temperatures (Fig. 11) investigated for both the catalyst samples prepared via sol-gel and hydrothermal methods showed that, an increased in annealing temperature resulted in a decrease in TiO_2_ surface area. This trend was similar for both catalysts prepared under the different preparation methods investigated. However, the decrease in surface area for catalysts samples prepared *via* sol-gel was observed to be drastic from 200 °C (∼182 m^2^/g) to 400 °C (∼62 m^2^/g). Comparably, the observed decrease in surface area as a function of annealing temperature was gradual for the catalyst samples prepared by hydrothermal method. More specifically, the as-synthesized TiO_2_ nanoparticles by hydrothermal method exhibited a surface area of 207 m^2^/g, which further decreased to ∼182 m^2^/g after annealing at 200 °C. A further rise in the calcining temperature from 200 °C to 400 °C, yielded a surface area of ∼126 m^2^/g, representing a two-fold increase in the surface area obtained over the TiO_2_ samples annealed at the same temperature. The rapid decrease in surface area of titania prepared by sol-gel as compared to that prepared using the hydrothermal method when the calcination temperature was increased might be attributed to the enhanced dehydroxylation of the titania samples prepared by sol-gel upon calcination possible due to the weak covalent Ti-O bonds.

**Fig. 12:**
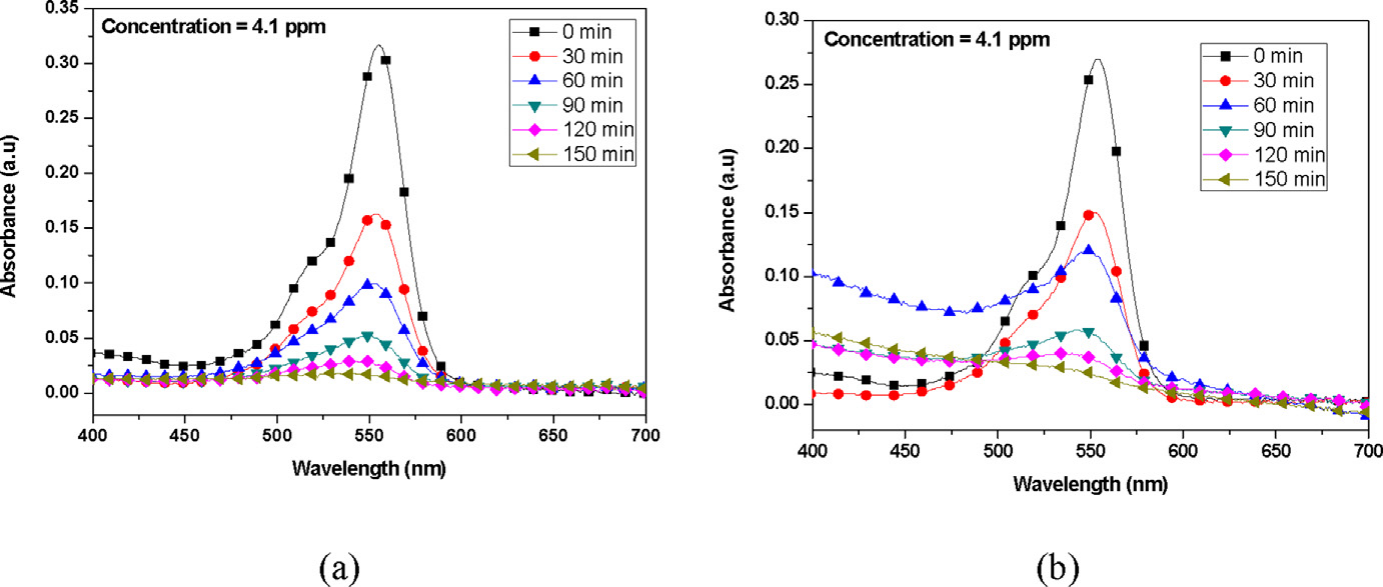
Absorption Spectrum of Rhodamine B dye degraded by (a) sol gel prepared catalyst and (b) Hydrothermal prepared catalyst.

At a characteristic wavelength of 554 nm, the absorbance peak was recorded at various exposure times for Rhodamine B dye solutions. Prior to the photocatalytic experiments, a control experiment with Rhodamine B dye solution without TiO_2_ catalyst showed negligible or no degradation when illuminated by ultraviolet light over 150 min exposure. Also, Rhodamine B dye solution with TiO_2_ catalyst without Ultraviolet light illumination also showed negligible or no degradation over a 150 min. This showed that both the TiO_2_ catalyst and ultraviolet light are required to initiate the photocatalytic reaction to cause the degradation of the dye solution. At an initial Rhodamine B dye concentration of 4 ppm the absorption spectrum was studied and reported as seen in Fig. 12. As can be seen from Fig. 12, it is obvious that the photodegradation rate of Rhodamine B by sol-gel approach was efficient compared to TiO_2_ synthesized by hydrothermal approach. The difference in efficiency may be attributed to the better activity between the cationic Rhodamine B dye and titania. Since only anatase phase was produced by sol-gel approach, photocatalysis was better than the hydrothermal method, that produced both rutile and anatase phases (Figs. 1 and 2). It is well known that anatase phase of TiO_2_ is most active and in terms of photo activity, anatase is more reactive than rutile because it has a larger surface area, higher electron-hole pair mobility, and improved surface hydroxyl density [37].

At a characteristic wavelength of 507 nm, the absorbance of Sudan III dye was monitored and evaluated as it was degraded by the prepared TiO_2_ catalyst. Sudan III dye exposed to UV light without any TiO_2_ catalyst showed no or negligible degradation over a 150 minutes' period. Also, the Sudan III dye with catalyst but not exposed to UV light showed no or negligible degradation.

**Fig. 13:**
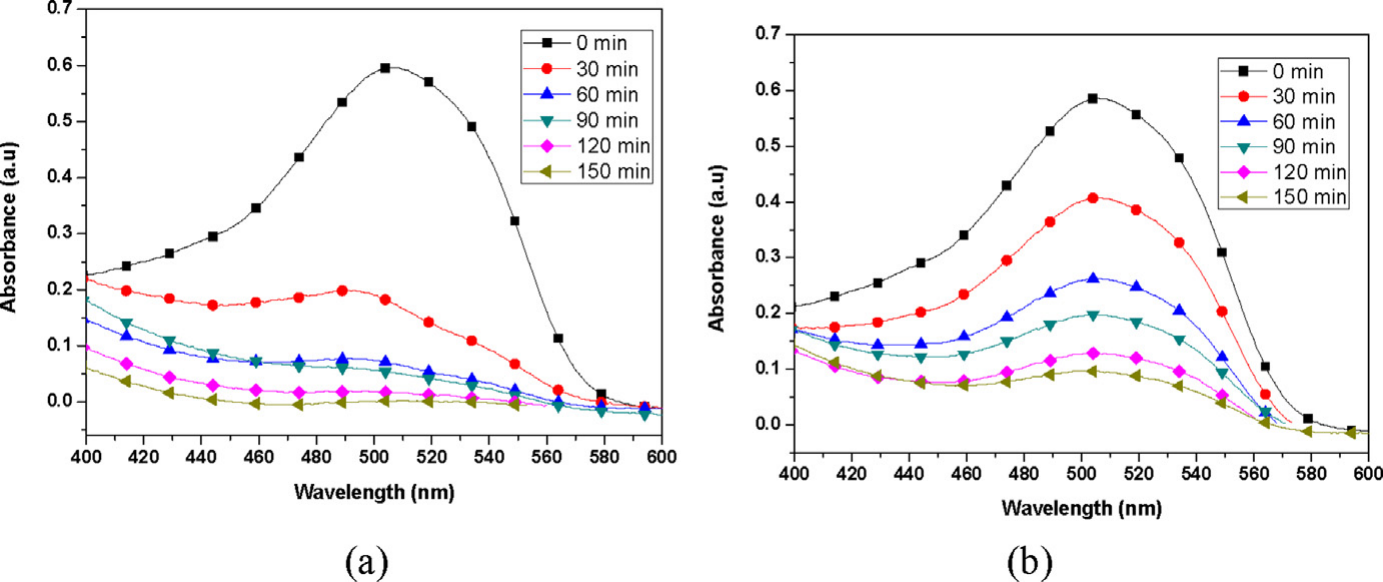
Absorption Spectrum of Sudan III dye degraded by (a) Hydrothermal prepared TiO_2_ catalyst, and (b) Sol gel prepared TiO_2_ catalyst.

At initial Sudan III dye concentration of 183.3 ppm the absorption spectrum was studied and reported as shown in Fig. 13. Surprisingly, Sudan III dye degradation by TiO_2_ prepared by hydrothermal method was rather higher than for sol-gel approach. After 150 minutes of irradiation, titania synthesized by hydrothermal approach gave 100 % degradation whilst that by sol-gel approach gave 83 % degradation. This can be attributed to presumably, the large specific surface area (Fig. 11) of the hydrothermally synthesized TiO_2_, which produced mainly anatase, phase titania between 450 °C–500 °C (Fig. 1). Again, crystallite domain size of TiO_2_ synthesized by hydrothermal approach was 15 nm for temperature range of 450 °C–500 °C. This therefore means a large surface area was available for reaction when compared to the TiO_2_ synthesized by sol-gel approach. Even though, anatase phase is reactive than rutile phase, it appears the synergistic effect between anatase and rutile phases, and the neutral surface charge of Sudan III, effectively result in higher photodegradation of Sudan III by hydrothermally synthesis method compared to the sol-gel approach.

At initial Sudan III dye concentration of 183.3 ppm, there was a negligible degradation of the Sudan III dye solution when the dye solution concentration was increased to 2008.7 ppm while keeping the catalyst loading constant. This is because the active sites on the catalyst which is available for the catalysis reaction is very vital for the decomposition to take place, however, as the dye concentration increases while the catalyst amount is kept constant, less active sites become available for the effective photodecomposition reactions. As the concentration of the dye were raised, the colour of the solution turns out to be deeply coloured; decreasing the path length of light traversing the solution and thereby allowing fewer photons to reach the surface of the catalyst. As a result, the generation of degradation species as well as limitation of the radicals reduced further the light path length leading to negligible photo-degradation [38].

In addition, pH affects the rate of adsorption and subsequent degradation. The influence of pH on dye photo degradation has extensively been studied elsewhere [9, 12, 39, 40]. It is well known that TiO_2_ has a point of zero charge (PZC) of 6.8. For acidic media (pH < PZC), TiO_2_ exists as positively charged catalyst. Conversely, titanium dioxide possesses a negatively charged surface in alkaline media (PH > PZC). Consequently, for a cationic dye like Rhodamine B, TiO_2_ photocatalysis would be much efficient because of the presence of hydroxyl radicals at the TiO_2_ active sites. In alkaline media, there is high surface hydroxyl anion density and therefore these hydroxyl anions can attract photo-excited electron from the valence band to produce more hydroxyl radicals. Hydroxyl radicals are effective for photocatalytic degradation of adsorbed species (such as of cationic dye) on TiO_2_ active sites. Therefore, TiO_2_ effectively degrades cationic dyes in basic media compared to neutral dyes. In this work, pH range of 3.5–4.0 was observed for the photocatalysis of Rhodamine B. This implies that positively charged holes are the radicals that mediated the photocatalytic process and not hydroxyl radicals. In the case of Sudan III experiments, pH range of 8–4 was observed. Since the former is a neutral dye, there is presumably less of its adsorption on the titania active sites, hence degradation would be less compared to cationic dye. This explains the relatively high photocatalysis of Rhodamine B compared to Sudan III, particularly for TiO_2_ synthesized by sol-gel method. Generally, increasing degradation time leads to the formation of more radicals [38]. Since the photocatalytic decomposition of the dye molecules happens on the surface of photocatalyst where radicals (•OH, •O_2_^−^, h^+^) are available for photocatalytic degradation, the former are capable of initiating bond cleavages in the adsorbed dye molecules on the surface of the catalyst.

For the photocatalytic decomposition of Rhodamine B Dye over TiO_2_ nanoparticles, the main products of decomposition are CO_2_ and H_2_O [41, 42].

## 4 Conclusions

Nanostructured TiO_2_ powders were efficaciously produced via the sol-gel and hydrothermal routes and characterized by X-ray Diffraction, BET, Fourier Transform Infrared spectroscopy, Raman Spectroscopy and Scanning Electron Microscopy methods. Analysis from the XRD and Raman confirmed that the synthesized particles were pure anatase phase TiO_2_ except the sample prepared by hydrothermal method and calcined at 600 °C which showed **15 %** rutile phase and **85 %** anatase phase. The synthesized catalysts were used in the photocatalytic degradation of Sudan III and Rhodamine B dyes at various durations of exposure to the Ultra Violet light. The photocatalytic activities of these nanostructures were estimated by determining the decrease in concentration of the respective dye solutions over time when TiO_2_ was used as the catalyst. It was demonstrated that Rhodamine dye and Sudan III dye were degraded within a short time. The sol gel prepared catalyst showed a higher photocatalytic activity in Rhodamine B dye solution and the hydrothermal prepared catalyst showed a higher photocatalytic activity in Sudan III dye solution. Typically, 94 % decomposition of Rhodamine B dye was observed when the sol-gel synthesized catalysts were used and Ultra Violet (UV) light irradiated for 150 minutes. However, for the same period of irradiation, 100 % decomposition of Sudan III dye was observed when the hydrothermally prepared catalysts were used. From these investigation, these produced nanostructured TiO_2_ powders have potential applications in the decomposition of a wide variety of dye contaminants.

## Declarations

### Author contribution statement

David Dodoo-Arhin: Conceived and designed the experiments; Analyzed and interpreted the data; Wrote the paper.

Frederick Paakwah Buabeng: Conceived and designed the experiments; Performed the experiments; Analyzed and interpreted the data; Contributed reagents, materials, analysis tools or data.

Julius M. Mwabora: Performed the experiments; Analyzed and interpreted the data.

Prince Nana Amaniampong, Henry Agbe, Emmanuel Nyankson, David Olubiyi Obada, Nana Yaw Asiedu: Analyzed and interpreted the data.

### Funding statement

This research did not receive any specific grant from funding agencies in the public, commercial, or not-for-profit sectors.

### Competing interest statement

The authors declare no conflict of interest.

### Additional information

No additional information is available for this paper.
